# Tracheotomy in a High-Volume Center During the COVID-19 Pandemic: Evaluating the Surgeon’s Risk

**DOI:** 10.1177/0194599820955174

**Published:** 2020-09-01

**Authors:** Arielle G Thal, Bradley A. Schiff, Yasmina Ahmed, Angela Cao, Allen Mo, Vikas Mehta, Richard V. Smith, Hillel W. Cohen, Thomas J. Ow

**Affiliations:** 1 Department of Otorhinolaryngology–Head and Neck Surgery, Montefiore Medical Center, Albert Einstein College of Medicine, The Bronx, New York, USA; 2 Department of Radiation Oncology, Montefiore Medical Center, Albert Einstein College of Medicine, The Bronx, New York, USA; 3 Department of Epidemiology and Population Health, Montefiore Medical Center, Albert Einstein College of Medicine, The Bronx, New York, USA; 4 Department of Otorhinolaryngology–Head and Neck Surgery and Department of Pathology, Montefiore Medical Center, Albert Einstein College of Medicine, The Bronx, New York, USA

**Keywords:** SARS-CoV-2, tracheotomy, personal protective equipment, PPE, COVID-19, coronavirus

## Abstract

**Objective:**

Performing tracheotomy in patients with COVID-19 carries a risk of transmission to the surgical team due to potential viral particle aerosolization. Few studies have reported transmission rates to tracheotomy surgeons. We describe our safety practices and the transmission rate to our surgical team after performing tracheotomy on patients with COVID-19 during the peak of the pandemic at a US epicenter.

**Study Design:**

Retrospective cohort study.

**Setting:**

Tertiary academic hospital.

**Methods:**

Tracheotomy procedures for patients with COVID-19 that were performed April 15 to May 28, 2020, were reviewed, with a focus on the surgical providers involved. Methods of provider protection were recorded. Provider health status was the main outcome measure.

**Results:**

Thirty-six open tracheotomies were performed, amounting to 65 surgical provider exposures, and 30 (83.3%) procedures were performed at bedside. The mean time to tracheotomy from hospital admission for SARS-CoV-2 symptoms was 31 days, and the mean time to intubation was 24 days. Standard personal protective equipment, according to Centers for Disease Control and Prevention, was worn for each case. Powered air-purifying respirators were not used. None of the surgical providers involved in tracheotomy for patients with COVID-19 demonstrated positive antibody seroconversion or developed SARS-CoV-2–related symptoms to date.

**Conclusion:**

Tracheotomy for patients with COVID-19 can be done with minimal risk to the surgical providers when standard personal protective equipment is used (surgical gown, gloves, eye protection, hair cap, and N95 mask). Whether timing of tracheotomy following onset of symptoms affects the risk of transmission needs further study.

The 2019 coronavirus pandemic (COVID-19), caused by severe acute respiratory syndrome coronavirus 2 (SARS-CoV-2), has affected >5 million Americans and claimed the lives of >161,000 as of August 10, 2020.^1^ While many patients will have mild disease, approximately 5% will become critically ill requiring intubation and prolonged mechanical ventilation.^2^ Prior to this pandemic, tracheotomy was routinely recommended for patients intubated for a prolonged period. In the setting of COVID-19, the decision to proceed with tracheotomy poses new questions due to the unclear risk-benefit ratio. One major consideration is the potential risk of transmission from the patient to the health care team, as this procedure can be a source of aerosolized virus during and after tracheotomy tube placement.

Previous reports documented an increased risk of SARS-CoV-2 transmission when operating on mucosal surfaces of the head and neck due to high viral loads in the upper aerodigestive tract.^3^ Entering and manipulating the airway of a patient harboring active SARS-CoV-2 virus poses obvious risks. In an effort to protect personnel involved in the procedure, protocols to decrease transmission during tracheotomy were created, and recommendations were published and widely circulated on the appropriate level of personal protective equipment (PPE).^2-4^ While some guidelines recommended the use of powered air-purifying respirators for any aerosol-generating procedure, others recommended standard airborne precautions, relying mostly on eye protection and N95 respirators. At the current time, literature has not materialized demonstrating that enhanced PPE offers improved protection from SARS-CoV-2. We report our experience performing tracheotomy with standard PPE for airborne precautions (N95 mask, face shield, hair net, gown, and gloves) in patients diagnosed with SARS-CoV-2. We describe the specific protective equipment used and safety measures taken and report the exposures and rate of infection among the tracheotomy surgical providers.

## Methods

A retrospective chart review was completed of all patients requiring prolonged intubation due to SARS-CoV-2 infection who underwent tracheotomy by the otolaryngology service at Montefiore Medical Center. The research was approved by the Institutional Review Board at the Albert Einstein College of Medicine. Following consent, providers in our department involved in tracheotomy were surveyed regarding the protective measures taken during tracheotomy, presence of COVID-19 symptoms, results of SARS-CoV-2 testing, and antibody status before and after involvement in tracheotomy procedures.

### Tracheotomy Procedure and Team

Institutional guidelines describing safe practices and PPE were created and circulated at the beginning of the COVID-19 pandemic (March 22, 2020). The otolaryngology team adhered to these guidelines throughout the study period. Tracheotomy was performed in the operating room (OR) or at the bedside in the intensive care unit (ICU) or general medical floor. Bedside procedures were generally favored to limit the risk of transmission during patient transport, to decrease the need for dedicated surgical staff, and to lower burden on OR time and personnel during the pandemic. OR procedures were carried out if the patient required other surgical procedures (eg, gastrostomy tube placement) or if the operating theater setting was deemed more appropriate by the attending surgeon. On the general medical floor, the procedural team included an attending surgeon, a resident surgeon, an anesthesiologist, and the patient’s nurse. In the ICU, the procedural team consisted of an attending surgeon, a resident surgeon, a respiratory therapist, the ICU nurse, and covering critical care staff, as needed, to administer medications. The number of people in the room was limited to only those necessary to execute the procedure, manage the endotracheal tube, and administer medications.

### Protective Measures

Our consultation team advocated for repeat SARS-CoV-2 polymerase chain reaction (PCR) testing prior to tracheotomy to assess risk of transmission. However, this was not mandated and was not consistently performed, as it did not change medical or procedural decision making. Standard PPE for airborne precautions were maintained for every tracheotomy performed during the pandemic, regardless of repeat test results. According to guidelines from the US Centers for Disease Control and Prevention, this included a surgical cap, face shield/eye protection, N95 mask, and surgical gown and gloves (some physicians used 2 sets of gloves). Protection was donned prior to entry into the room and removed prior to exiting. All surgeons had been educated via institutional information sessions and electronic notifications regarding proper donning-and-doffing procedures for PPE. Tracheotomy procedures were carried out in routine open fashion. The surgical team preferred an open approach over percutaneous methods due to theoretical safety advantages secondary to the degree of control of the airway throughout the procedure, without the need for confirmatory bronchoscopy. Specific to the COVID-19 pandemic, all patients were routinely paralyzed throughout the procedure, and suction and cautery were minimized to avoid aerosolization of viral particles. Some providers also used anticholinergics to decrease secretions and thus minimize suctioning requirements. At entry into the trachea, the ventilator was placed on stand-by to eliminate positive-pressure aerosolization of tracheal secretions. Positive-pressure ventilation was resumed after placement of the tracheotomy tube with inflation of the cuff. Placement was confirmed with the usual methods, including adequate inspiratory and expiratory tidal volumes and/or end tidal carbon dioxide monitoring.

### Data Collection

We completed a retrospective chart review of all tracheotomies performed by the otolaryngology service on patients admitted with symptoms of COVID-19 and a positive SARS-CoV-2 PCR test result. Dates of admission, intubation, and tracheotomy were obtained to assess time to tracheotomy. Dates and results of SARS-CoV-2 PCR testing were collected with attention paid to the timing in relation to tracheotomy. The involved surgeons (based on operative notes) were surveyed to assess the safety practices utilized, the presence of COVID-19 symptoms, and the result of SARS-CoV-2 antibody testing.

### Outcome Measures

The primary outcome was SARS-CoV-2 antibody positivity or symptomatic illness of the attending and house staff surgeons involved in the tracheotomy procedures. Antibody testing was made available by our institution and offered to all providers. It was performed according to the presence of SARS-CoV-2 symptoms or desire of the provider to undergo testing; there were no specific guidelines or mandates to obtain testing at our institution during the study period. Data were not collected for respiratory therapists, anesthesiologists, nurses, or ICU staff involved, as these providers were highly varied across dates, locations, and operative settings.

### Statistical Analyses

Data for this study are largely descriptive and presented as whole numbers, proportions or percentages, and mean estimates, where appropriate, with ranges included as necessary. A swimmer’s plot was constructed to graphically represent the number of COVID-19 tracheotomy exposures for each surgeon. To estimate the likelihood of transmission of SARS-CoV-2 based on the data in this study, we constructed 95% CIs using the exact formula (Clopper-Pearson).^5^

## Results

### Case Series

Thirty-six tracheotomies in patients diagnosed with SARS-CoV-2 were identified and reviewed for this study. The locations were at the bedside in 30 of 36 (83.3%), of which 6 (20%) were done on the medical floors and 24 (80%) in the ICU. One of the tracheotomies done in the OR was in conjunction with thyroidectomy for compressive goiter. The average time from hospital admission for COVID-19 symptoms to tracheotomy was 31 days, with a range of 20 to 61 days. For patients with an immediate perioperative positive SARS-CoV-2 test result, the average time from hospital admission for COVID-19 symptoms to tracheotomy was 28 days, with a range of 20 to 38 days.

### SARS-CoV-2 Viral Swab Testing

**Figure 1.**
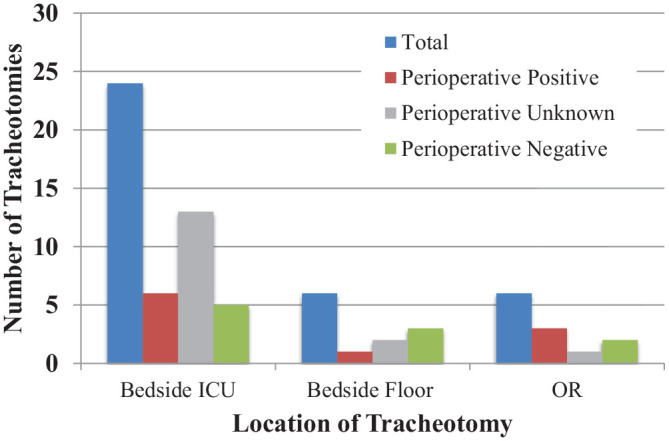
Number of patients with COVID-19 receiving tracheotomy stratified by repeat SARS-CoV-2 testing status at the time and location of tracheotomy. ICU, intensive care unit; OR, operating room.

All patients included in this review tested positive for SARS-CoV-2 during hospitalization. Ten patients (27.8%) had an immediate preoperative positive test result. This included any patient with a positive test result both before and after tracheotomy or within 72 hours prior to tracheotomy. Ten patients (27.8%) had a negative preoperative test result. The remaining 16 did not undergo SARS-CoV-2 PCR testing in the immediate preoperative setting. Two patients had a positive test result 5 days prior to tracheotomy; 1 patient, a positive test result 10 days prior; 3 patients, a negative test result 3 to 5 days after tracheotomy; 4 patients, a negative test result 7 to 10 days after; and 6 patients, no test result within 10 days before or after tracheotomy. The location of tracheotomies and the immediate perioperative testing status for these procedures are summarized in **Figure 1**.

### Surgeon Results

**Figure 2.**
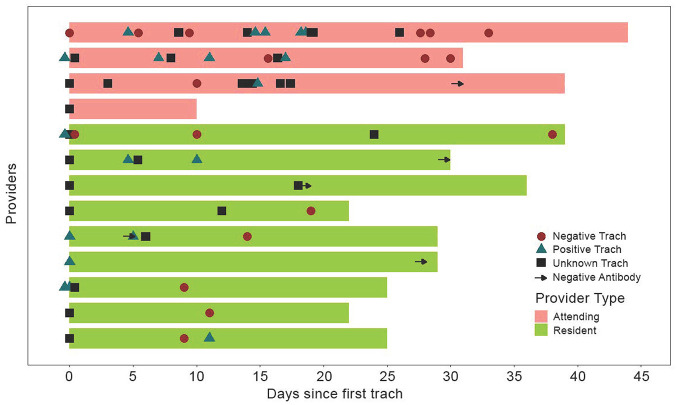
Swimmer’s plot demonstrating the risk timeline for each tracheotomy provider, starting with time of exposure from first tracheotomy performed to the time of data collection. Each subsequent tracheotomy exposure, as well as the timing of antibody testing (arrow), is displayed along each timeline for each provider. Blue triangle, tracheotomy performed on a patient who remained SARS-CoV-2 positive on perioperative testing; red circle, tracheotomy performed on a patient whose status converted to SARS-CoV-2 negative on perioperative testing; black square, tracheotomy performed on a patient for which perioperative SARS-CoV-2 testing was not done.

All tracheotomies were performed by 1 of 4 attending surgeons, who performed 16, 10, 9, and 1 tracheotomies. There were 11 resident surgeons involved; 2 of them were seropositive after developing COVID-19 prior to performing any tracheotomy for patients with SARS-CoV-2. Because they were hypothetically not at risk of contracting SARS-CoV-2 from the tracheotomy exposure, these 2 individuals were excluded from the final analysis. Overall, 4 attending surgeons and 9 house staff were involved in tracheotomies and included in the study. After exclusion of the previously mentioned providers, there were 65 exposures during tracheotomy included in our study: 19 to patients with an immediate preoperative positive SARS-CoV-2 test result, 18 to patients with an immediate preoperative negative SARS-CoV-2 test result, and 28 to patients with unknown SARS-CoV-2 testing status at time of tracheostomy. All surgeons followed the institutional recommendations for PPE and safety measures listed in **Table 1**. There were zero COVID-19 symptoms or seroconversions as a result of involvement in tracheotomy among the surgeons involved. Characteristics and exposures for the surgical providers are summarized in **Table 2**. **Figure 2** presents a swimmer’s plot to display the exposures of each surgeon and to provide a timeline for the period at risk from exposures to the time of our study survey for each participant.

**Table 1.** Equipment Employed and Specific Risk-Mitigating Measures for Tracheotomy During the COVID-19 Pandemic.

Protective equipment	Protective measures
**Personal protective equipment** • Surgical cap • Face shield/eye protection • N95 mask • Surgical gown • Surgical gloves **Tracheotomy supplies** • Tracheotomy tray (instrument set) • Lightbox and headlight • Monopolar electrocautery unit • Sterile drapes	• Bedside procedure when possible to decrease risk of transmission during transfer to the operating room • Negative-pressure room when possible • Paralytic initiated prior to incision • Glycopyrrolate to decrease secretions (variable) • Essential team members in room only • Preoperative timeout, including verification of appropriate personal protective equipment • Limited use of suction/cautery • Ventilation on stand-by at entry into trachea • Appropriate donning/doffing of personal protective equipment

**Table 2.** Characteristics and COVID-19/SARS-CoV-2 Status of Tracheotomy Surgical Team.^a^

	Surgeon (n = 4)	House staff (n = 9)
Provider		
Age	45.5 (40-53)	30 (25-34)
Male	4	2
Female	0	7
No. of tracheotomies done per surgeon		
Overall	9.5 (1-16)	3.5 (1-6)
With immediate perioperative positive COVID-19	2.5 (0-5)	1 (0-2)
No. of providers		
With COVID-19 symptoms	0	0
Tested for SARS-CoV-2 PCR test		
Positive	0	0
Negative	1	2
Not tested	3	7
Tested for antibodies		
Positive	0	0
Negative	2	7
Not tested	2	2
Likely to have contracted COVID-19 (aggregate)		
Yes	0	0
No	4	9

Abbreviation: PCR, polymerase chain reaction.

a Values are presented as median (range) or No.

To estimate the likelihood of transmission given our results, we constructed a 95% CI using the exact formula (Clopper-Pearson).^5^ Based on 36 tracheotomy procedures, if the real population rate of transmission was 0.05 and the outcome was defined as any transmission occurring in any of the surgical providers present at the tracheotomy, the 95% CI would be 0.005 to 0.179. If the real population rate was 0.03, the 95% CI would be 0.001 to 0.149. We repeated this analysis using 65 surgical provider exposures, with a potential outcome of 1 transmission per exposure. Based on hypothetical population transmission rates of 0.05 and 0.03, the respective 95% CIs were 0.011 to 0.134 and 0.004 to 0.106. These results suggest that whether the tracheotomy procedure is used as the unit of analysis or the surgical provider exposure, there would be roughly a <2.5% probability of observing zero transmissions if the true population rate was as low as 0.03.

## Discussion

At the onset of the COVID-19 pandemic in the United States, there were varying recommendations regarding the performance of tracheotomy given the risk of transmission to providers and the lack of evidence on which patients would benefit from it. Based on data from prior experience with highly communicable respiratory diseases, such as SARS, it was known that health care providers involved in endotracheal intubation or tracheotomy had a higher risk of viral contraction.^6^ There were also reports of providers being infected despite proper PPE, which led to some recommendations for enhanced PPE, including helmets, hoods, and powered air-purifying respirators.^7^ Early experience with SARS-CoV-2 in China indicated that health care workers involved in tracheotomy had a greater risk (odds ratio, 4.15) of viral contraction than those who did not.^8^ This led to further recommendations regarding enhanced PPE when performing tracheotomy. In addition, recommendations against performing tracheotomy at all in patients who were SARS-CoV-2 positive were also widely accepted due to the risk of contraction by providers.^2^ As the otolaryngology community adjusted to these recommendations, the number of SARS-CoV-2 patients at our institution dramatically increased and persisted for several weeks. As a result, a careful approach to tracheotomy was necessary to optimize patient care and disposition and to properly allocate resources. After review of the existing literature, the decision was made to proceed with tracheostomy with standard PPE instead of powered air-purifying respirators. Despite initial concern, no transmission events were documented from patients to providers via standard SARS-CoV-2 PPE.

All providers in our study wore N95 masks, face shields, surgical caps, surgical gowns, and gloves. Arguably, the N95 mask was the most important piece of PPE among these items. N95 masks are effective at filtering 99.5% of particles >0.75 µm.^9^ The SARS-CoV-2 viral particle diameter is 0.06 to 0.12 µm,^10^ and it travels within aerosol particles, which are >1 µm. The N95 mask therefore protects providers against 99.5% of viral particles of SARS-CoV-2. Data regarding the variance in efficacy among brands are inconclusive. There was no standardized brand, style, or type worn by providers in our study. The masks worn were distributed by the institution, which varied throughout the study period. Based on our experience, an infectious transmission of virus from affected patient to provider during tracheotomy did not occur. Thus, we support the use of standard PPE during tracheotomy for patients testing positive for SARS-CoV-2.

One element that may have protected the providers in this study was the time from initial diagnosis to tracheotomy. The average time to tracheotomy in our cohort from hospital admission for COVID-19 symptoms was 28 days, with a range of 20 to 38 days in patients with an immediate perioperative positive SARS-CoV-2 test result. The timing of tracheotomy should balance the risk of transmission to the surgical team and the benefit of tracheostomy for the patient. Patient outcomes are not reported in this study; therefore, we are unable to comment on the ideal timing of tracheotomy.

The virulence of SARS-CoV-2 and the natural history of viral loads and viral shedding among patients with severe COVID-19 disease are not fully understood. The majority of patients in our study tested negative for SARS-CoV-2 on immediate perioperative PCR or were not tested at the time of tracheotomy. Recent data suggest that even after upper respiratory tract samples become negative, lower respiratory tract samples may remain positive for up to 39 days.^11^ The correlation between positive results by quantitative PCR viral testing and infectivity is still being investigated. Recent studies showed that positive viral cultures were more common among patients with a positive SARS-CoV-2 PCR test result who had lower cycle threshold values (number of cycles required to obtain a detectable amount of viral RNA from viral swabs), suggesting that patients who test positive have higher viral loads and high virulence. This study deduced that patients who test negative on PCR with higher cycle threshold values may not be excreting infectious viral particles and are thus less likely to put those in contact with them at risk of contraction.^12^ Further data suggest that patients affected by SARS-CoV-2 are infectious beginning 2.3 days prior to symptom onset, with infectivity peaking at 0.7 days before symptom onset and rapidly declining within 8 days following symptom onset. The majority of our patients underwent tracheotomy outside this window. However, these data were derived from patients with moderate forms of the disease. In addition, we concede that this is likely affected by the use of antivirals, anti-inflammatories, antibiotics, and immunomodulators,^13^ which were not measured in our review. However, all patients in our study did receive hydroxychloroquine and at least 3 days of steroids during admission prior to tracheotomy, per standard protocols recommended by the infectious disease service at our institution during the early COVID-19 surge in New York in March and April 2020.

The previously discussed data support decreased risk of transmission in patients with negative viral swabs. However, several patients in our study had a SARS-CoV-2–positive PCR test result at the time of tracheotomy, and most providers performed at least 1 tracheotomy on 1 of these patients (and, in most cases, several). Despite this, no providers exhibited COVID-19 symptoms or seroconverted. In addition, all of the patients included in our study had the severe form of the disease, requiring intubation and mechanical ventilation. Data suggest that patients with the severe form of the disease have a higher viral load that decreases more slowly than those with mild disease.^14^ We believe that our report is representative of the exposure risks at most high-volume centers in the midst of the COVID-19 epidemic.

Despite our detailed report, our study has several limitations. Since we do not have precise data of SARS-CoV-2 viral load in the respiratory tracts at the time of tracheotomy for each case, we cannot clearly quantify the risk in our cohort. We also were not able to collate data for risk of transmission to other patients and health care staff on the basis of these procedures or posttracheostomy care. Furthermore, we were not able to assess risk of transmission to the nursing staff, respiratory therapists, or anesthesia staff, as these providers varied at each operation, though anecdotally these rates were very low based on ongoing discussions with our hospital staff. The majority of procedures were done in single-patient rooms, not all of which were negative-pressure rooms. Negative-pressure rooms were preferred to prevent the airborne particles inside the infected patient’s room from entering the rest of the unit and infecting other patients or providers. However, because of the extensive number of patients with COVID-19 at our institution, negative-pressure rooms were often allocated to nonintubated patients who were at risk of spreading virus to others, and in large part negative-pressure rooms were not available for tracheotomy procedures. Future studies may aim to evaluate whether negative-pressure rooms decrease the risk of transmission to other health care workers, as we are not able to comment on this in this study. In addition, now that we better understand the role of tracheotomy in the setting of the COVID-19 pandemic, future studies should consider assessing the risk to all health care providers during posttracheostomy care.

All of our providers continued to wear standard PPE for routine tracheostomy care and tracheostomy tube changes. We performed tracheostomy tube changes only in the setting of air leak causing respiratory distress or when downsizing tracheotomy tubes for decannulation. The providers in our cohort were relatively young without significant comorbidities; this may have affected the presence of symptoms related to the exposure during tracheotomy. While several of these providers had antibody-negative testing after involvement, some reported only the lack of symptoms as the outcome measure. The CIs calculated for the transmission rate with 65 surgical staff exposures did not account for lack of statistical independence of those exposures. A larger study across multiple institutions would help to definitively conclude that our findings are widely generalizable.

Despite these limitations, we believe that this review provides data to support minimal risk of contraction to the surgical team when performing tracheotomy in patients with SARS-CoV-2, using standard PPE for airborne precautions. Further studies are needed to quantify this risk, and we recommend routine review and adherence to the most up-to-date safety measures.

## Conclusion

Our data support that tracheotomy for patients with SARS-CoV-2 can be done with minimal risk to the surgical providers when standard PPE is used (surgical gown, gloves, eye protection, hair cap, and N95 mask). In a large-volume setting with a relatively high number of tracheotomies, there was zero rate of transmission as a result of involvement in the procedure. Each center should adopt the safest measures available when preparing for high-risk procedures in the COVID-19 era, and we recommend corroborative reports before these measures are widely accepted.
